# Using Wavelet Packet Transform for Surface Roughness Evaluation and Texture Extraction

**DOI:** 10.3390/s17040933

**Published:** 2017-04-23

**Authors:** Xiao Wang, Tielin Shi, Guanglan Liao, Yichun Zhang, Yuan Hong, Kepeng Chen

**Affiliations:** State Key Laboratory of Digital Manufacturing Equipment and Technology, Huazhong University of Science and Technology, Wuhan 430000, China; wangxiao1989@hust.edu.cn (X.W.); tlshi@hust.edu.cn (T.S.); zhangyc@hust.edu.cn (Y.Z.); hongyuan@hust.edu.cn (Y.H.); ckphust@hust.edu.cn (K.C.)

**Keywords:** wavelet packet transform, surface characterization, roughness analysis

## Abstract

Surface characterization plays a significant role in evaluating surface functional performance. In this paper, we introduce wavelet packet transform for surface roughness characterization and surface texture extraction. Surface topography is acquired by a confocal laser scanning microscope. Smooth border padding and de-noise process are implemented to generate a roughness surface precisely. By analyzing the high frequency components of a simulated profile, surface textures are separated by using wavelet packet transform, and the reconstructed roughness and waviness coincide well with the original ones. Wavelet packet transform is then used as a smooth filter for texture extraction. A roughness specimen and three real engineering surfaces are also analyzed in detail. Profile and areal roughness parameters are calculated to quantify the characterization results and compared with those measured by a profile meter. Most obtained roughness parameters agree well with the measurement results, and the largest deviation occurs in the skewness. The relations between the roughness parameters and noise are analyzed by simulation for explaining the relatively large deviations. The extracted textures reflect the surface structure and indicate the manufacturing conditions well, which is helpful for further feature recognition and matching. By using wavelet packet transform, engineering surfaces are comprehensively characterized including evaluating surface roughness and extracting surface texture.

## 1. Introduction

The real surface has been defined in ISO (International Organization for Standardization) as a set of features that physically exist and separate the entire work piece from the surrounding medium [1]. The surface of a work piece has significant influence on its functional performance, and surface metrology becomes more and more vital for describing the surface processing quality. Surface characterization means the breakdown (filtering process) of the surface geometry into basic components usually based on the functional requirements [2]. Research on filtering methods for surface metrology has been studied for decades from a 2RC (second-order resistor-capacitor circuit) filter, Gaussian filter to wavelet filter and morphological filter. The 2RC filter is a nonlinear filter suffering from phase distortion [3]. Gaussian filter is the most widely used filter, adopted as the standard filtering technique by the standards committees [4]. Morphological filtration is carried out by performing morphological operations on the input signal. It has a drawback of being time-consuming for a large data set [5,6]. Wavelet, an attractive mathematical tool, can describe the surface with variable resolution. By comparing the signal with a series of template functions, researchers can use wavelets to analyze different frequency components with variable window sizes [7,8]. Chen et al. first introduced wavelet analysis to surface characterization in 1999 [9], which could evaluate surface roughness more accurately than traditional methods. In the 2000s, wavelet transform together with optimization algorithms were utilized for surface roughness analysis, showing its feasibility and applicability [10,11,12,13]. Jiang et al. applied the second and third generation wavelet model for surface analysis. Both the models were proved to be more effective than the conventional filtering methods. [14,15,16,17,18]. A dual-tree complex wavelet transform was used for surface analysis by Zeng et al. and Ren et al. showing very good amplitude transmission characteristics and high frequency resolution [19,20,21]. Wang et al. and Grzesik et al. combined wavelet analysis and fractal dimension for surface roughness evaluation [22,23], and the self-similarity of the surface could be detected. Rosenboom et al. and Doshi et al. applied wavelet analysis for surface description, defect detection and texture classification [24,25,26,27]. Yang et al. used wavelet filter for generating surface with confocal laser scanning microscope (CLSM) [28], demonstrating a powerful tool for eliminating image noise. Wavelet transform provides flexible time-frequency resolution, whereas it suffers from a relatively low resolution in high frequency regions, leading to difficulty in differentiating high frequency transient components [7]. Wavelet packet transform (WPT), an extension of wavelet transform which can further decompose the detailed information of the signal in high frequency regions, has been used for feature extraction and texture segmentation. Its performance of feature extraction was better than that of discrete wavelet transform [29,30]. Kim et al. presented an optimal algorithm based on WPT for surface quality characterization, improving the performance of texture classification without recursive calculation [31]. Makieta applied wavelet packet strategy for assessing milled surfaces. He provided criteria for choosing the basic wavelet and evaluated the surface roughness and waviness [32]. WPT [33] and one-dimensional (1D) wavelet transform [34] were also used to extract surface features for surface roughness evaluation based on an artificial neural network, where the statistical features were correlated with the surface roughness parameter *Ra*. Wavelet packet neural networks were also used for surface texture classification [35,36].

In this paper, WPT is applied to multi-resolution analysis on the high frequency components of real surface topography. Roughness parameters are calculated and surface textures are extracted to characterize the surface. A roughness specimen and three real engineering surfaces are then analyzed in detail. Profile and areal roughness parameters are calculated to quantify the characterization results and compared with those measured by a profile meter. The relations between roughness parameters and noise are analyzed in detail. This paper is organized as follows: Section 2 provides the theories of wavelet transform and WPT. The processing procedures of simulations and experiments are presented in Section 3. Section 4 exhibits the results and discussion about the simulated profiles and engineering surfaces. The conclusions are given in Section 5.

## 2. Theory

Wavelet transform is the local transform of the time and frequency with good localization features in the spatial domain and frequency domain. By translation and dilation, a mother wavelet, defined as $\psi_{s,\tau }\left(t\right)=\frac{1}{\sqrt{s}}\left(\frac{t-\tau }{s}\right)$, can generate wavelets [7]. The discrete scale parameter and translation parameter are expressed as $s=s_{0}^{j}$ and $\tau =k\tau_{0}s_{0}^{j}$. We then obtain the corresponding family of the base wavelet, $\psi_{j,k}\left(t\right)=\frac{1}{\sqrt{s_{0}^{j}}}\psi \left(\frac{t-j\tau_{0}s_{0}^{j}}{s_{0}^{j}}\right)$. The discrete wavelet transform of a given signal *x*(*t*) is obtained as $Wt\left(j,k\right)=\frac{1}{\sqrt{2^{j}}}\int_{-\infty }^{\infty }x\left(t\right)\psi \left(\frac{t-k2^{j}}{2^{j}}\right)dt$. Two-dimensional (2D) wavelets decomposition can be represented by horizontal, vertical, and diagonal wavelet functions.

Compared with wavelet transform, WPT has relatively high resolution in the high frequency region. As an extension of wavelet transform, WPT keeps many merits of wavelet transform, such as multi-resolution and multi-scale. Wavelet packet is defined by

(1) $$\left\{ \begin{array}{l} W_{2n}\left(x\right)=\sqrt{2}\sum_{k}h_{k}W_{n}\left(2^{p}x-k\right)\\ W_{2n+1}\left(x\right)=\sqrt{2}\sum_{k}g_{k}W_{n}\left(2^{p}x-k\right) \end{array}\right.$$

where *k* is a localization parameter, *p* is a scale index, *h* is a low pass filter, and *g* is a high pass filter. The function *W*_0_(*x*) can be identified with the scaling function *φ* and *W*_1_ with the basic wavelet function *ψ*. The corresponding 2D wavelet packet filters can be expressed as

(2) $$\left\{ \begin{array}{l} h_{LL}\left(k,l\right)=h\left(k\right)*h\left(l\right)\\ h_{HL}\left(k,l\right)=g\left(k\right)*h\left(l\right)\\ h_{LH}\left(k,l\right)=h\left(k\right)*g\left(l\right)\\ h_{HH}\left(k,l\right)=g\left(k\right)*g\left(l\right) \end{array}\right..$$

**Figure 1:**
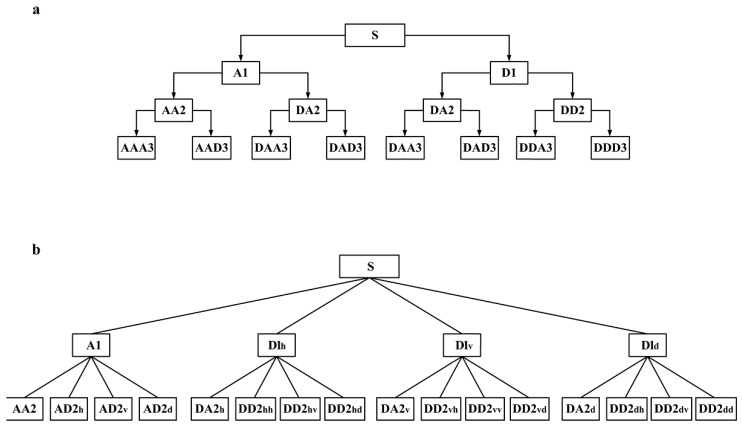
(**a**) Procedure of 1D wavelet packet transform (WPT); (**b**) procedure of 2D WPT.

Figure 1a schematically illustrates the decomposition procedure of 1D WPT, in which the signal is decomposed into three levels with eight sub-bands in total. Figure 1b shows the procedure of 2D WPT for surface topography.

The bi-orthogonal wavelets including two different sets of wavelets and scaling functions are usually used for the reconstruction and decomposition of surface topography [7], whose symmetry ensures that they have linear phase characteristics, important for surface texture analysis. They have been used for surface profile filtering in manufacturing process monitoring and diagnosing [7,37]. In this study, a bi-orthogonal 6.8 wavelet is used as the mother wavelet, and we then calculate the roughness parameters and extract the surface textures for assessing the engineering surfaces.

## 3. Simulations and Experiments

WPT is performed on a simulated 2D surface profile composed of a sinusoid line (the surface waviness) and a random signal (the roughness). Here, the roughness is obtained by the high frequency component reconstructed by using WPT. Border deformation is exhibited. Smooth padding, a border extension method, is chosen to avoid boundary deformation.

Then, surface roughness analysis is implemented on a surface roughness specimen with right-angle steps. Measurement is conducted by a confocal laser scanning microscope (CLSM, VK-X200, Keyece Co., Ltd., Osaka, Japan), where a 20× objective is used to capture the height topography of the surface, in which noisy points along the edge of the steps always exist. A wavelet threshold denoising method is implemented on the measured surface, and the border processing (BP) is carried out to eliminate the border deformation. By applying WPT to the surface topography, we obtain the reference surface and roughness surface together with the profile and areal parameters. For comparison, a profile meter (PM, talysurf PGI830, Taylor Hobson Ltd., Leicester, UK) is used to provide a criterion. The sample length is 3.0 mm and the evaluation length is 15.0 mm to evaluate profile roughness. The sample area and evaluation area are both 9 mm^2^ to estimate the areal roughness. The relations between the surface roughness parameters and noise are also analyzed by simulation, where four surfaces with the same shape and size in different levels of noise are used for surface roughness evaluation. The noise gradually increases from level-0 to level-3. We separate the surface topography by WPT and calculate the roughness parameters together with the relative errors (the parameters of the surfaces with level-1, level-2, and level-3 noise vs. those of the surface with level-0 noise). WPT as a smooth filter is also carried out on the high frequency components for extracting the surface texture. After obtaining the filtered surface topography, we search for the extreme points in a 3 × 3 template as the candidate feature points and confirm the texture feature points by setting a threshold. A milled surface, a turned surface and a grinding surface are further analyzed for verification.

## 4. Results and Discussion

**Figure 2:**
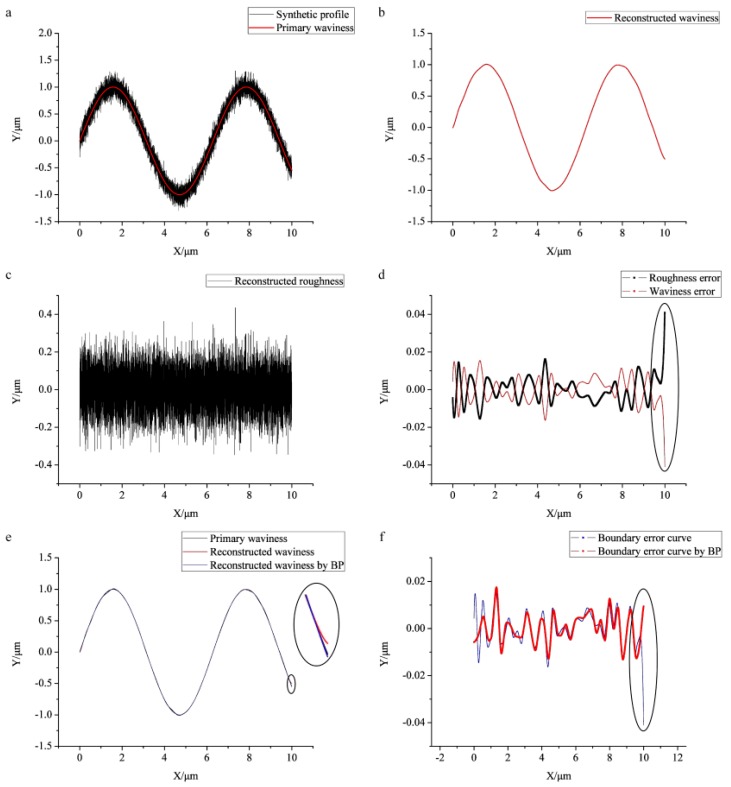
The simulated results for the decomposition of the surface profile: (**a**) the simulated profile; (**b**) the waviness line separated by WPT; (**c**) the reconstructed roughness line by WPT; (**d**) the errors between the primary waviness (roughness) and the reconstructed waviness (roughness) by using WPT; (**e**) the primary waviness, reconstructed waviness, reconstructed waviness by border processing (BP); (**f**) the errors of the reconstructed waviness with or without BP relative to the primary waviness.

Figure 2a displays the simulated surface profile including waviness and roughness. Figure 2b exhibits the separated waviness (also the filtering reference line), and Figure 2c shows the reconstructed roughness by WPT. The error curves are given in Figure 2d, where the red line represents the differences between the reconstructed waviness and primary waviness, and the black line denotes the errors between the reconstructed roughness and primary roughness, as it can be found that the waviness and roughness obtained by WPT match well with the primary waviness and roughness. At the end of two curves, an outlier marked in a circle indicates that border deformation appears. Figure 2e presents the primary waviness (black line), reconstructed waviness (red line), and reconstructed waviness after BP (blue line). The inset of the enlarged drawing illustrates that the reconstructed waviness by BP almost keeps the same as the primary waviness, whereas some deviations exist between the primary waviness and reconstructed waviness without BP. The error curves given in Figure 2f exhibit the effect of BP clearly, in which the red line represents the errors between the primary waviness and reconstructed waviness by BP, which has a relatively smaller absolute value at the end than the blue line (the errors between the primary waviness and reconstructed waviness without BP). Obviously, the boundary deformation is eliminated.

**Figure 3:**
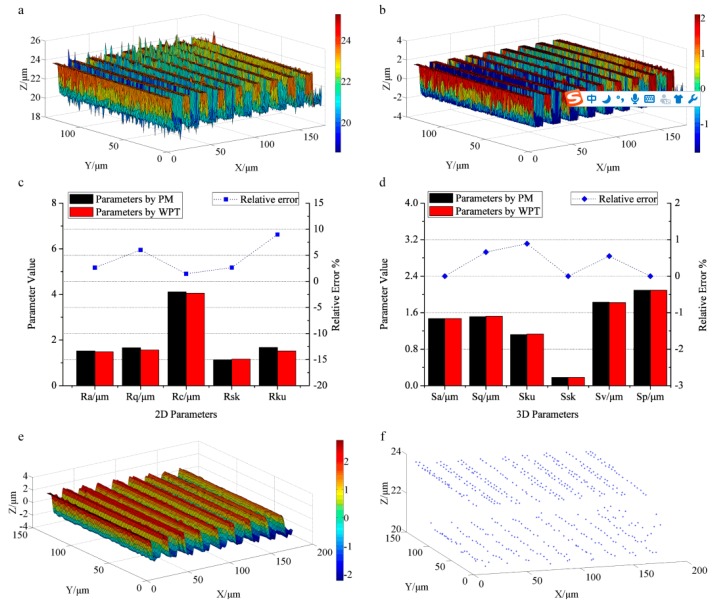
The characterization results of the roughness specimen: (**a**) the primary surface topography measured by the confocal laser scanning microscope (CLSM); (**b**) the roughness topography obtained by WPT; (**c**) the profile roughness parameters and their relative errors calculated by WPT and the profile meter (PM); (**d**) the areal roughness parameters and their relative errors obtained by WPT and PM; (**e**) the smoothed surface topography by WPT; (**f**) the extracted 490 feature points of surface texture.

The characterization results of the roughness specimen with right-angle steps are presented in Figure 3. Figure 3a shows the primary surface topography captured by CLSM, where the color bar denotes the height information. The noisy points are obviously visible along the right angle edge, which will influence the generation of roughness surface seriously. The filtered result is illustrated in Figure 3b, in which the noisy points are removed. The de-noising procedure contributes to generating the roughness surface accurately. Figure 3c displays the characterization results of the 2D profile roughness, including profile parameters *R_a_* (the arithmetical mean deviation), *R_q_*(the root mean square deviation), *R_ku_* (the kurtosis), *R_sk_* (the skewness), and *R_c_* (the mean height of profile elements) [38]. The left vertical axis represents the value of the parameters and the right axis denotes the percentage of the relative errors. The roughness parameters calculated by WPT are exhibited as the red bars, and the black bars signify the parameters measured by PM for comparison. The calculated results for *R_a_*, *R_q_*, *R_c_*, *R_sk_*, and *R_ku_* by WPT are 1.48 μm, 1.56 μm, 4.05 μm, 1.16, and 1.52, similar to the measurement results by PM (1.52 μm, 1.66 μm, 4.11 μm, 1.13, and 1.67, correspondingly). The blue scatter dot line represents the relative errors of the calculation results by WPT vs. those by PM, where the relative errors of *R_a_*, *R_q_*, *R_c_*, and *R_sk_* are all rather small (2.63%, 6.02%, 1.46% and 2.65%). *R_ku_* obtained by WPT appears the largest deviation compared with that by PM (the relative error approaching 8.98%). *R_ku_* represents the kurtosis of the assessed profile and measures the sharpness of the probability density function of the ordinate values. Both the measurements of the right-angle steps structure by CLSM and PM may introduce noisy points. These points with a relatively large value will become isolated valleys or peaks and influence *R_ku_* seriously [38], resulting in the inaccuracy of the characterization. Figure 3d displays the areal surface roughness parameters together with the relative errors. The areal parameters of *S_a_* (the arithmetical mean height), *S_q_* (root mean height), *S_ku_* (the kurtosis), *S_sk_* (the skewness), *S_v_* (the maximum pit height), and *S_p_* (the maximum peak height) [39] calculated by WPT (1.47 μm, 1.52 μm, 1.13, 0.18, 1.82 μm, and 2.09 μm) match well with those measured by PM (1.47 μm, 1.51 μm, 1.12, 0.18, 1.83 μm, and 2.09 μm, correspondingly). The relative errors are all less than 1%, indicating the reliability of WPT for the evaluation of surface roughness.

The filtered surface by applying WPT to the high frequency components (roughness components) is shown in Figure 3e. It keeps the basic structure of the original surface topography. The extracted 490 feature points displayed in Figure 3f express the typical surface texture containing the top textures and the bottom textures, which suggest the basic direction of the textures and are helpful for further feature recognition and matching. Therefore, WPT can also be used for extracting surface texture by decomposing the high frequency components of the surface topography.

**Figure 4:**
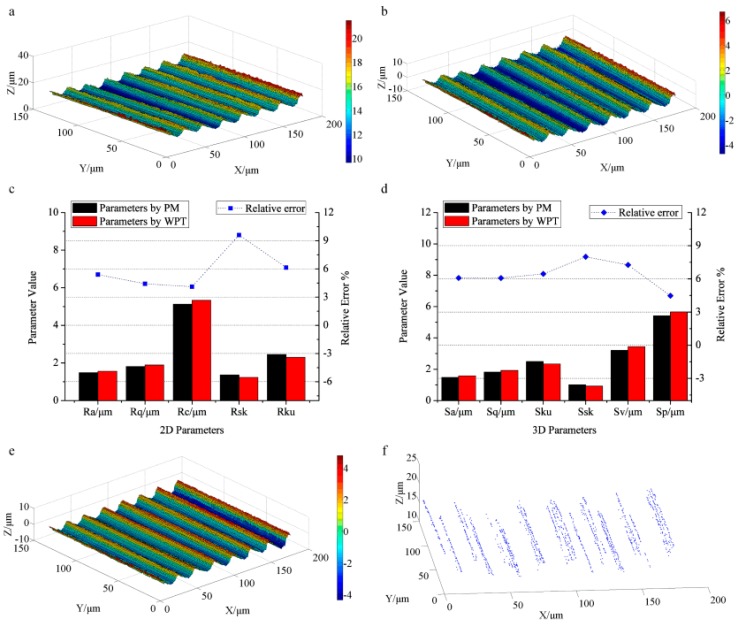
The analyzed results of the milled surface: (**a**) the primary surface topography by CLSM, (**b**) the roughness topography obtained by WPT; (**c**) the profile roughness parameters and their relative errors calculated by PM and WPT; (**d**) the areal roughness parameters together with relative errors obtained by WPT and PM; (**e**) the smoothed surface topography by WPT; (**f**) the extracted 1371 feature points of surface texture.

Figure 4 displays the analyzed results of the milled surface. Figure 4a illustrates the surface topography, and Figure 4b displays the roughness surface reconstructed by WPT. The profile roughness parameters and their relative errors are exhibited in Figure 4c. The values of *R_a_*, *R_q_*, *R_c_*, *R_sk_*, and *R_ku_* calculated by WPT are 1.56 μm, 1.89 μm, 5.33 μm, 1.23, and 2.30. Those measured by PM (1.48 μm, 1.81 μm, 5.12 μm, 1.36, and 2.45) are used for comparison. It can be seen that the profile roughness parameters obtained by WPT approximate to those measured by PM. The relative errors are 5.41%, 4.42%, 4.10%, 9.56%, and 6.12%, respectively, indicating that the characterization results obtained by WPT agree well with those by PM. The largest deviation appears in *R_sk_*, which measures the asymmetry of the probability density function of the ordinate values, strongly influenced by the noise in the measurements. The corresponding areal roughness parameters together with the relative errors are shown in Figure 4d. *S_a_*, *S_q_*, *S_ku_*, *S_sk_*, *S_v_*, and *S_p_* by WPT are 1.57 μm, 1.92 μm, 2.33, 0.92, 3.44 μm, and 5.65 μm. For comparison, the results by PM are 1.48 μm, 1.81 μm, 2.49, 1.00, 3.21 μm, and 5.41 μm. The relative errors of *S_a_*, *S_q_*, *S_ku_*, and *S_p_* (6.08%, 6.07%, 6.43%, and 4.43%) are less than 7%. However, the difference of *S_v_* reaches 7.16%, and the relative error of *S_sk_* even approaches 8.00%. *S_v_* measures the largest surface valley depth with a sampling region. *S_sk_* evaluates the asymmetry of the probability density function of the ordinate values. Noisy points introduced in the measurements will result in large deviations of *S_v_* and *S_sk_*.

The filtered surface topography is illustrated in Figure 4e. It retains the main surface structure. There are 1371 feature points extracted from Figure 4e as displayed in Figure 4f, clearly exhibiting the top textures and the bottom tool marks, which reflect the basic texture structure of the whole surface. The periodic textures suggest the direction of the surface texture and help to predict the surface performance. The evaluation of surface roughness and the extraction of surface texture are successfully achieved by analyzing the high frequency components of surface based on WPT.

**Figure 5:**
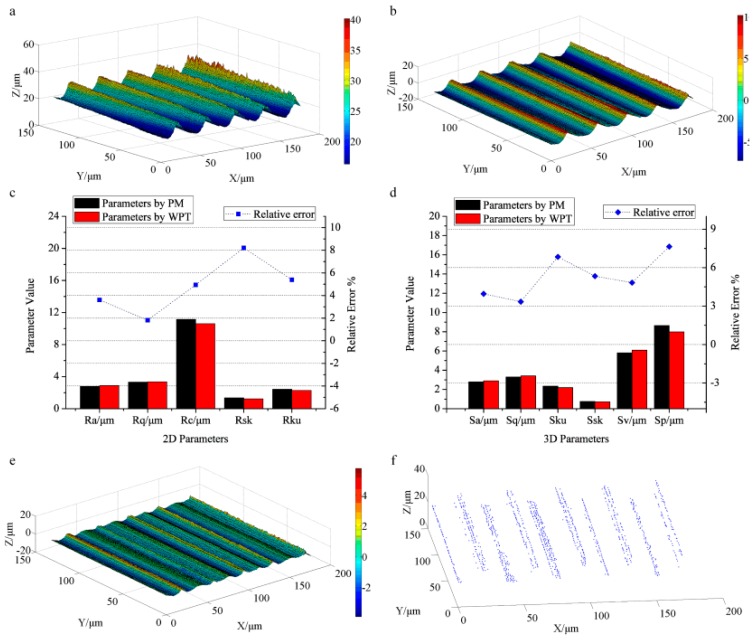
The evaluation results of the turned surface: (**a**) the primary surface topography by CLSM; (**b**) the roughness topography obtained by WPT; (**c**) the profile roughness parameters and their relative errors calculated by PM and WPT; (**d**) the areal roughness parameters and their relative errors obtained by WPT and PM; (**e**) the smoothed surface topography by WPT; (**f**) the extracted 1126 feature points of surface texture.

The characterization results of the turned surface are also presented in Figure 5. Figure 5a,b display the primary surface and filtering results. The results of the profile roughness parameters are shown in Figure 5c, in which the calculated *R_a_*, *R_q_*, *R_c_*, *R_sk_*, and *R_ku_* by WPT (2.87 μm, 3.35 μm, 10.59 μm, 1.23, and 2.28) approximate the measurement results by PM (2.77 μm, 3.29 μm, 11.14 μm, 1.34, and 2.41). The corresponding relative errors are 3.61%, 1.82%, 4.94%, 8.21%, and 5.39%, signifying the characterization results of the 2D profile roughness by WPT match well with those by PM. Figure 5d exhibits the characterization results of areal roughness. The parameter values of *S_a_*, *S_q_*, *S_ku_*, *S_sk_*, *S_v_*, and *S_p_* by WPT are 2.89 μm, 3.40 μm, 2.18, 0.71, 6.08 μm, and 7.98 μm, similar to the results by PM (2.78 μm, 3.29 μm, 2.34, 0.75, 5.80 μm, and 8.64 μm). The relative errors of *S_a_*, *S_q_*, *S_ku_*, *S_v_*, and *S_sk_* (3.96%, 3.34%, 6.84%, 4.83%, and 5.33%) are less than 7%. Only *S_p_* reaches 7.64%, probably caused by noisy points.

Figure 5e exhibits the smooth surface by applying WPT to the high frequency components. It is also a periodic structure much flatter than the roughness component as shown in Figure 5b. Figure 5f displays the extracted surface texture, which contains 1126 feature points presenting the tool marks clearly including the top ridge textures and bottom valley textures. The reconstructed surface typical textures not only indicate the direction of the surface texture but also help to recognize and classify surface texture.

**Figure 6:**
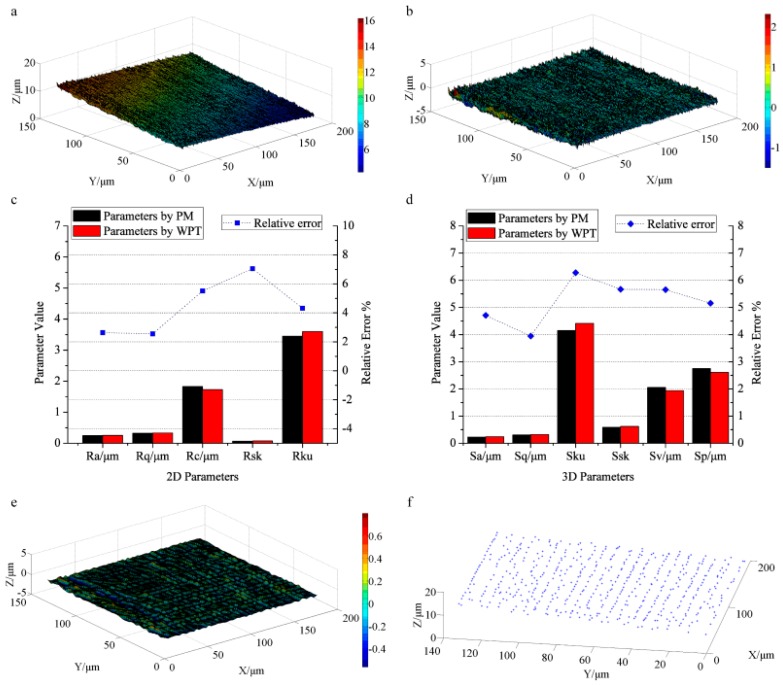
The characterized results of the grinding surface: (**a**) the primary surface topography by CLSM; (**b**) the roughness topography obtained by WPT; (**c**) the profile roughness parameters and their relative errors calculated by PM and WPT; (**d**) the areal roughness parameters and their relative errors obtained by WPT and PM; (**e**) the smoothed surface topography by WPT; (**f**) the extracted 532 feature points of surface texture.

The characterized results of the grinding surface are presented in Figure 6. The original surface captured by CLSM is shown in Figure 6a. Figure 6b provides the roughness surface. The evaluation results of the profile roughness are given in Figure 6c. The obtained profile roughness parameters (*R_a_*, *R_q_*, *R_c_*, *R_sk_*, and *R_ku_*) are 0.25 μm, 0.33 μm, 1.72 μm, 0.073, and 3.60. For comparison, the measured results by PM are 0.24 μm, 0.32 μm, 1.82 μm, 0.068, and 3.45, respectively. As a result, the relative errors of *R_a_*, *R_q_*, *R_c_*, and *R_ku_* are 2.63%, 2.54%, 5.51%, and 4.31%. Only *R_sk_* reaches 7.04%. Figure 6d exhibits the evaluated results of the areal roughness, where the calculated results of *S_a_*, *S_q_*, *S_ku_*, *S_sk_*, *S_v_* and *S_p_* by WPT (0.24 μm, 0.31 μm, 4.41, 0.62, 1.94 μm, and 2.60 μm) approximate the results by PM (0.23 μm, 0.30 μm, 4.15, 0.58, 2.05 μm, and 2.75 μm). The relative errors (4.71%, 3.94%, 6.28%, 5.67%, 5.65%, and 5.16%) are all smaller than 7% suggesting the reliability of WPT for assessing surface roughness.

Figure 6e shows the filtered surface components, in which we extract 532 feature points as displayed in Figure 6f. Since the surface is fabricated by grinding tool and the surface is rather flat without periodic texture, the extracted feature points only suggest the direction of the texture without layered features. Thus, by decomposing the high frequency components of the surface topography based on WPT, the engineering surfaces are comprehensively assessed including surface roughness evaluation and surface texture extraction.

**Figure 7:**
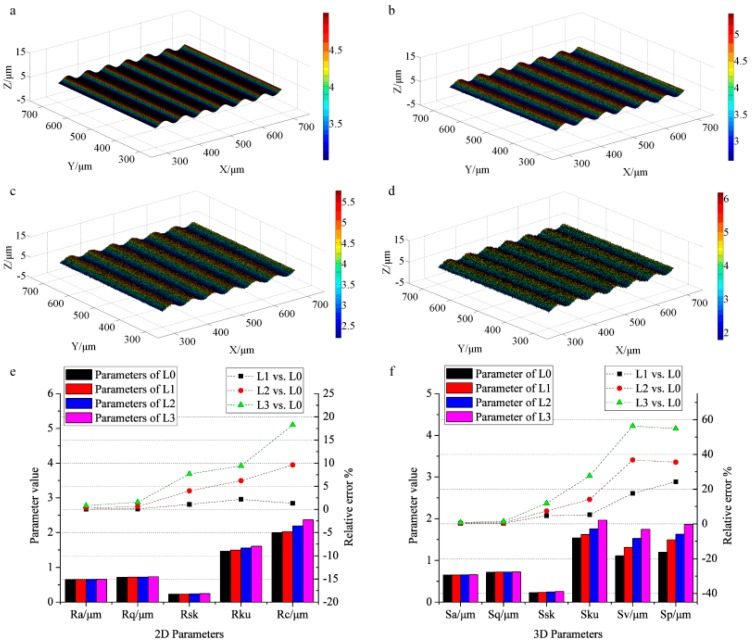
The analyzed results of the relations between parameters and noise: the surface topography with level-0 noise (**a**), level-1 noise (**b**), level-2 noise (**c**), and level-3 noise (**d**); (**e**) the profile roughness parameters and their relative errors; (**f**) the areal roughness parameters and their relative errors.

Figure 7a–d show the simulated surfaces with level-0, level-1, level-2, and level-3 noise, respectively. Figure 7e displays the evaluated results of the profile roughness of every surface (the black, red, blue, and pink bars represent the results of the surfaces with level-0, level-1, level-2, and level-3 noise, respectively, and the black, red, and green dot lines signify the relative errors of the surfaces with level-1, level-2, and level-3 noise vs. those of the surface with level-0 noise, respectively). The results (*R_a_*, *R_q_*, *R_sk_*, *R_ku_*, and *R_c_*) of the surface with level-0 (0.65 μm, 0.72 μm, 0.23, 1.47, 2.00 μm), level-1 (0.65 μm, 0.72 μm, 0.23, 1.50, 2.03 μm), level-2 (0.65 μm, 0.72 μm, 0.24, 1.56, 2.19 μm), and level-3 (0.6558 μm, 0.73 μm, 0.25, 1.60, 2.36 μm) noise are calculated. Accordingly, the relative errors (*R_a_*) of the surfaces with level-1, level-2, and level-3 noise vs. that of the surface with level-0 noise are 0.046%, 0.31%, and 0.78%. The relative errors of *R_q_* (0.097%, 0.61%, and 1.57%), *R_sk_* (1.09%, 4.00%, and 7.65%), *R_ku_* (2.18%, 6.20%, and 9.41%), and *R_c_* (1.32%, 9.60%, and 18.24%) are also illustrated as the dot lines. As can be found, the skewness (*R_sk_*), kurtosis (*R_ku_*), and the mean height (*R_c_*) increase drastically with the noise increasing from level-0 to level-3, explaining that the parameters (*R_sk_*, *R_ku_* and *R_c_*) are influenced by noise much stronger than *R_a_* and *R_q_*. This leads to relatively large deviations of *R_sk_*, *R_ku_*, and *R_c_* by WPT and PM.

The evaluated results of the areal roughness are displayed in Figure 7f. The parameters *S_a_*, *S_q_*, *S_sk_*, *S_ku_*, *S_v_*, and *S_p_* of the surface with level-0 noise are 0.65 μm, 0.72 μm, 0.24, 1.54, 1.11 μm, and 1.20 μm. The corresponding results of the surfaces with level-1 (0.6523 μm, 0.72 μm, 0.24, 1.62, 1.31 μm, 1.40 μm), level-2 (0.65 μm, 0.72 μm, 0.25, 1.75, 1.52 μm, and 1.63 μm), and level-3 (0.66 μm, 0.73 μm, 0.26, 1.96, 1.74 μm, and 1.86 μm) noise are also presented. Accordingly, the relative errors (*S_a_*) of the surfaces with level-1, level-2, and level-3 noise vs. that of the surface with level-0 noise are 0.26%, 0.54%, and 0.81%. The relative errors of *S_q_* (0.36%, 0.70%, and 1.33%), *S_sk_* (4.67%, 7.38%, and 11.88%), *S_ku_* (5.24%, 14.09%, and 27.64%), *S_v_* (17.62%, 36.81%, and 56.40%), and *S_p_* (24.25%, 35.56%, and 54.93%) are also illustrated as the dot lines. Obviously, *S_sk_*, *S_ku_*, *S_v_* and *S_p_* significantly increase when the noise increases from level-0 to level-3, whereas *S_a_* and *S_q_* increase slowly. Therefore, the skewness (*S_sk_*), kurtosis (*S_sk_*), the maximum pit (*S_v_*) and peak (*S_p_*) heights are strongly influenced by noise, leading to the relatively large deviations in the experimental results.

## 5. Conclusions

In this paper, we evaluate the surface roughness and extract the typical texture using WPT for a number of common surface finishes. A CLSM is used to capture the surface topography, and the high frequency components of the surface topography are analyzed in detail. Smooth border padding is introduced to eliminate the border deformation, and the de-noise process is executed to obtain the roughness surface accurately. A simulated 2D surface profile is tested by using WPT, and the reconstructed roughness coincides with the original ones. Then, a roughness specimen together with three engineering surfaces (a milled surface, a turned surface, and a grinding surface) is used for further verification. Profile roughness parameters (*R_a_*, *R_q_*, *R_c_*, *R_ku_*, and *R_sk_*) and areal roughness parameters (*S_a_*, *S_q_*, *S_v_*, *S_p_*, *S_ku_*, and *S_sk_*) are calculated and compared with the measurement results by PM. It can be found that the roughness parameters obtained by WPT agree well with the measurement results by PM. The relations between the roughness parameters and noise are analyzed by simulation for explaining the relatively large deviations occurring in the results. The extracted textures clearly exhibit the surface structure and the basic tool marks, also being helpful for texture classification and surface performance estimation. These proved that WPT is feasible and capable for comprehensively characterizing a number of common surface finishes, including evaluating surface roughness and extracting surface texture.
